# Nutritional Enhancement of Health Beneficial Omega-3 Long-Chain Polyunsaturated Fatty Acids in the Muscle, Liver, Kidney, and Heart of Tattykeel Australian White MARGRA Lambs Fed Pellets Fortified with Omega-3 Oil in a Feedlot System

**DOI:** 10.3390/biology10090912

**Published:** 2021-09-14

**Authors:** Shedrach Benjamin Pewan, John Roger Otto, Robert Tumwesigye Kinobe, Oyelola Abdulwasiu Adegboye, Aduli Enoch Othniel Malau-Aduli

**Affiliations:** 1 Animal Genetics and Nutrition, Veterinary Sciences Discipline, College of Public Health, Medical and Veterinary Sciences, Division of Tropical Health and Medicine, James Cook University, Townsville, QLD 4811, Australia; shedrach.pewan@my.jcu.edu.au (S.B.P.); john.otto@jcu.edu.au (J.R.O.); robert.kinobe@jcu.edu.au (R.T.K.); 2 National Veterinary Research Institute, Private Mail Bag 01 Vom, Plateau State, Nigeria; 3 Public Health and Tropical Medicine Discipline, College of Public Health, Medical and Veterinary Sciences, Division of Tropical Health and Medicine, James Cook University, Townsville, QLD 4811, Australia; oyelola.adegboye@jcu.edu.au

**Keywords:** MARGRA lamb, omega-3 fatty acids, *Longissimus thoracis et lumborum* muscle, kidney, heart, liver, meat quality, oil, whole grain

## Abstract

**Simple Summary:**

The problem addressed in this research was the possibility of enhancing the nutritional value and health beneficial omega-3 long-chain fatty acid content of lamb and its edible components. The aims and objectives were to evaluate the omega-3 contents of muscle, liver, kidney, and heart of lot-fed Tattykeel Australian White lambs of the MARGRA brand, in response to dietary supplementation with or without omega-3 oil fortified pellets. The findings demonstrate that the inclusion of omega-3 oil in feedlot diets of lambs enhances the human health beneficial omega-3 long-chain polyunsaturated fatty acid profiles of edible muscle tissue and organs without compromising meat quality or shelf life. These results are valuable to society because of increased functionality, health benefits, micro-marbling, tender, mouth-melting taste, and high-end eating quality experience of MARGRA lamb tissues and organs.

**Abstract:**

The aim of this research was to evaluate the nutritional enhancement of omega-3 long-chain polyunsaturated fatty acid (n-3 LC-PUFA) composition of edible lamb *Longissimus thoracis et lumborum* muscle, heart, kidney, and liver in response to dietary supplementation of lot-fed lambs with or without omega-3 oil fortified pellets. The hypothesis tested was that fortifying feedlot pellets with omega-3 oil will enhance the human health beneficial n-3 LC-PUFA composition of edible lamb muscle tissue and organs. Seventy-five Tattykeel Australian White lambs exclusive to the MARGRA brand, with an average body weight of 30 kg at six months of age, were randomly assigned to the following three dietary treatments of 25 lambs each, and lot-fed as a cohort for 47 days in a completely randomized experimental design: (1) Control grain pellets without oil plus hay; (2) Omega-3 oil fortified grain pellets plus hay; and (3) Commercial whole grain pellets plus hay. All lambs had *ad libitum* access to the basal hay diet and water. Post-slaughter fatty acid composition of the *Longissimus thoracis et lumborum* muscle, liver, kidney, and heart were determined using thee gas chromatography–mass spectrophotometry technique. Results indicated significant variations (*p* < 0.05) in fatty acid profiles between tissues and organs. Omega-3 oil fortified pellets significantly (*p* < 0.05) increased ≥C20 n-3 LC-PUFA (C20:5n-3 eicosapentaenoate, EPA + C22:5n3 docosapentaenoate, DPA + C22:6n3 docosahexanoate DHA); C18:3n-3 alpha-linolenate, ALA; C18:2 conjugated linoleic acid, CLA; total monounsaturated fatty acids, MUFA; polyunsaturated fatty acids, PUFA contents; and reduced the ratio of omega-6 to omega-3 fatty acids in all lamb organs and tissues without impacting shelf-life. The findings demonstrate that the inclusion of omega-3 oil in feedlot diets of lambs enhances the human health beneficial omega-3 long-chain polyunsaturated fatty acid profiles of edible muscle tissue and organs without compromising meat quality.

## 1. Introduction

Functional foods are among the fastest-growing markets in developed countries where the average consumer prefers omega-3 enrichment with information about the food’s production process [1]. Functional foods can influence satiety and a healthier lifestyle [2]. The main strategies for creating healthier and functional foods with increased satiety include the modification of dietary fat, fiber, and sugar compositions [2]. Ansorena and Astiasarán [3] provided insights into the methods of modifying the formulations of fresh, cooked, and fermented meat products in order to increase omega-3 fatty acid content without modifying animal diets. The fortification of functional beef burgers with microencapsulated cod liver oil [4], algal and wheat germ oil emulsions [5] are examples of methods for enriching foods with omega-3 fatty acids.

The demand for high quality meat is on the increase as consumer preferences for edible animal-based protein sources shift toward eating quality with increased human health benefits. Meat, an essential component of the human diet, is rich in nutrients including protein, fatty acids, iron, zinc, copper, selenium, and B-complex vitamins [6,7,8]. Omega-3 long-chain polyunsaturated fatty acids (n-3 LC-PUFA) are essential fatty acids that play diverse roles in human health and disease prevention. They include the following longer chain derivatives of alpha-linolenic acid (ALA, C18:3n-3): Eicosapentaenoic (EPA, C20:5n-3), docosapentaenoic (DPA, C22:5n-3), and docosahexaenoic (DHA, C22:6n-3) acids. EPA + DPA + DHA are known to promote intellectual development in infancy, relieve inflammation, boost the immune system, reduce incidences of cardiovascular diseases, some cancers, diabetes, allergies, behavioral disorders, and sustain retinal functions [9,10,11,12]. However, humans, like all mammals, cannot synthesize n-3 LC-PUFA because they are unable to produce Δ12 and Δ15-desaturase enzymes [13], hence, rely on dietary sources like leafy vegetables, oilseeds, nuts, eggs, and seafood, especially fish and crustaceans [10,14], edible marine algae, bacteria, fungi, diatoms, fruits, and herbs [15], to meet their daily n-3 LC-PUFA requirements. Oilseeds commonly used in human diets include rapeseed [16] and soybean [17], while seed oils from waste food by-products such as tomato [18] and citrus [19] are cheap animal feed sources that can enhance healthy fatty acid composition.

Fatty acid composition influences the nutritive value and organoleptic traits of meat including tenderness, flavor, and juiciness [20]. The fatty acid content of meat can be affected by the animal production system [21,22], breed or genotype [23,24], gender [25], age at slaughter [26], liveweight [27], level of fatness [20], type of muscle and feed. In lamb [28], cattle, swine, and poultry [29], it has been suggested that dietary manipulation can be utilized to improve the fatty acid content and nutritional value of meat that more closely meets nutritional guidelines. However, due to extensive rumen microbial biohydrogenation in ruminants, dietary polyunsaturated fatty acids (PUFA) are converted to saturated fatty acids (SFA), absorbed in the small intestine, and deposited in edible tissues (muscles), products (milk), and organs (liver, kidney and heart), thereby causing more health challenges to consumers [30,31]. Ruminant meat research is aimed at reducing saturated fatty acids and increasing the proportion of health-beneficial n-3 LC-PUFA [31]. Therefore, dietary supplementation with rumen-protected plant and fish-based n-3 LC-PUFA oil, forages, and concentrates containing bioactively enriched microalgae [32] are some of the steps taken by livestock farmers to improve the nutritional and health values of meat.

To the current knowledge of the authors of this research, there is presently no published literature on n-3 LC-PUFA metabolism in the *Longissimus thoracis et lumborum* muscle, heart, kidney, and liver of lot-fed Tattykeel Australian White (TAW) MARGRA lambs in response to dietary supplementation with omega-3 oil. The research reported in this present study intends to fill this knowledge gap. It was hypothesized that *fortifying feedlot pellets with omega-3 oil will enhance the human health beneficial n-3 LC-PUFA composition of edible lamb muscle tissue and organs*. Therefore, the primary objective of this study was to evaluate and compare the fatty acid profiles in the tissues and organs of TAW lambs raised in a feedlot production system in response to dietary supplementation with or without fortification with omega-3 oil.

## 2. Materials and Methods

### 2.1. Animals, Dietary Treatments, and Experimental Design

This lamb finishing feeding trial was conducted at the Crown Agriculture’s feedlot facility at Borenore, New South Wales, Australia, from April to June 2019. Borenore is located at latitude 33°19′S and longitude 149°04′E with an elevation of 3024 feet above sea level and average annual temperature of 11.7 °C (53.0 °F) and rainfall of 939.8 mm (37.0 inches). The feedlot was an automated facility in a well-ventilated covered building with concrete floors, density of five square meters per head with all the feeding troughs equipped with installed sensors capable of immediate data capture of each lamb’s ear tag identification, entry and exit times, body weight, feed intake, and other vital parameters. These data are automatically recorded, electronically cloud-stored, and directly downloadable into Excel spreadsheets and transmitted when required. Seventy-five Tattykeel Australian White lambs exclusive to the MARGRA brand, with an average body weight of 30 kg at six months of age, were randomly assigned to the following three dietary treatments of 25 lambs each, and lot-fed as a cohort for 47 days after a 14-day adaptation period in a completely randomized experimental design: (1) Control grain pellets without oil plus hay; (2) omega-3 oil fortified grain pellets plus hay; and (3) commercial whole grain pellets plus hay. All lambs had *ad libitum* access to the basal hay diet and water. The nutrient composition of the supplementary and basal diets is presented in Table 1.

At the end of the feeding trial, the lambs were conveyed during the cool hours of the day to the Gundagai Meat Processing Plant, New South Wales, Australia, held in lairage and fasted overnight. The lambs were humanely sacrificed as a single mob in line with Meat Standards Australia guidelines and industry best practice standards. The carcasses were subjected to medium voltage electrical stimulation before being trimmed and dressed [33]. The liver, kidney, and heart were sampled immediately following evisceration, vacuum-sealed in labelled bags, and stored at −20 °C pending fatty acid evaluation. All carcasses were held in the chiller room for 24 h at 4 °C and a sample of the *Longissimus thoracis et lumborum* muscle tissue was taken between the 12th and 13th ribs for fatty acid analysis.

### 2.2. Feed Sample Processing and Nutrient Composition Analysis

Supplementary and basal feed samples were oven-dried for three days at 60 °C, cooled, and ground to pass through a 1 mm sieve using a laboratory mill (Thomas Model 4 Wiley^®^ Mill; Thomas Scientific, Swedesboro, NJ, USA). Dry matter and ash percentages were determined using the AOAC standard laboratory analytical techniques [34]. Neutral detergent (NDF) and acid detergent (ADF) fiber percentages were determined using an Ankom Fiber Analyzer (ANKOM2000; ANKOM Technology, Macedon, NY, USA). Nitrogen content was determined using a Thermo Finnigan EA 1112 Series Flash Elemental Analyzer (Thermo Finnigan, Poway, CA, USA) and the values were multiplied by 6.25 to provide the expected crude protein (CP) percentage. Ether extract (EE) was analyzed employing an ANKOM^XT15^ fat/oil extractor (ANKOM Technology, Macedon, NY, USA).

### 2.3. Fatty Acid Analysis

Fatty acid analysis of feed, muscle tissue, liver, kidney, and heart samples was carried out at the Commonwealth Scientific and Industrial Research Organization (CSIRO), Food Nutrition and Bio-based Products, Oceans and Atmosphere Laboratory, Hobart, Tasmania, Australia. The gas chromatography–mass spectrophotometry total lipids and muscle phospholipids extraction procedures of Malau-Aduli et al. [35,36] based on an amended Bligh and Dyer technique [37], were utilized for fatty acid composition analysis where total lipids in 1 g of un-homogenized muscle tissue samples were extracted overnight. The original phase was a single-phase overnight extraction utilizing CHCl_3_:MeOH:H_2_O (1:2:0.8 *v*/*v*). The second segment involved phase separation with the addition of CHCl_3_:saline Milli-Q H_2_O (1:1 *v*/*v*) followed by rotary evaporation of the lower chloroform phase at 40 °C to acquire total lipids. The extracted cumulative lipids were separated into lipid classes by thin-layer chromatography (TLC) using 100 mL of the lipid extract reconstituted in n-hexane. The extract was marked onto silica gel G plates (200 × 200 × 0.25 mm^3^) using a micropipette. The TLC plate was developed in an acetone/petroleum ether (1:3 vol/vol) solvent system in a tank comprising a few crystals of butylated hydroxytoluene (BHT) to hinder oxidation. Triacylglycerols, cholesterol, and free fatty acids migrated, while phospholipids remained at the origin of the plate. The phospholipids were scraped off the plate into clean screw-capped test tubes for transmethylation and eventual computation of the lipid conversion factor (LCF) of 0.912 based on g fatty acids/g total lipids (0.083 for phospholipids, 0.829 for triacylglycerols, and 0% for cholesterol since cholesterol does not have any fatty acids). An aliquot from each total lipid extract was utilized for transmethylation with MeOH:CHCl_3_:HCl (10:1:1 *v*/*v*) for two hours at 80 °C. Fatty acid methyl esters (FAME) were extracted thrice using n-hexane:CHCl_3_ (4:1 *v*/*v*). A known concentration of an internal standard (C19:0) was added in a 1500 µL vial encompassing the extracted FAME. The FAME was analyzed on a 7890B gas chromatograph (Agilent Technologies, Palo Alto, CA, USA) furnished with an Equity^TM^ -1 fused 15 m silica capillary column with 0.1 mm internal diameter and 0.1 µm film thickness (Supelco, Bellefonte, PA, USA), a flame ionization sensor, a split/splitless injector, and an Agilent Technologies 7683 B Series autosampler. The gas chromatograph settings were splitless mode injection; carrier gas He; original oven temperature 120 °C and then increased to 270 °C at flow rates of 10 °C/min and to 310 °C at 5 °C/min. The Agilent Technologies ChemStation software (Palo Alto, CA, USA) was used to measure fatty acid peaks. The fatty acid identities were established using a Finnigan Thermoquest GCQTM GC/MS fitted with an on-column injector and Thermoquest Xcalibur software (Austin, TX, USA) as described in detail by Miller et al. [38]. Fatty acid percentages were calculated as follows: FA% = [(individual fatty acid area) × (100)]/(sum total area of fatty acids). Fatty acid contents were calculated as follows: FA mg/100 g = (Total lipid) × (LCF [0.912]) × ([%FA]/100) × 1000, where 0.912 was the resultant lipid conversion factor [39]. FA contents were presented in mg/100 g tissue as per Food Standards of Australia and New Zealand recommendations.

### 2.4. Statistical Analyses

Data analysis was performed as a completely randomized design using R statistical software version 3.6.3 [40]. Statistical inference was based on a 5% level of significance. Summary statistics of fatty acids composition were presented as means and standard deviations. The effect of dietary treatment was statistically analyzed separately using one-way analysis of variance (ANOVA) in the general linear model (GLM) procedure to investigate the fatty acid profile differences in the muscle, liver, kidney, and heart of the TAW lambs.

The model utilized was:

$$FA_{ij}=\mu +Feed_{i}+\epsilon_{ij}$$

where *FA* is the fatty acid composition; *μ* is the overall mean response; $Feed_{i}$ is the effect due to the *i*th treatment (*i* = 1 to 3; control, omega-3, MSM whole grain); and $\epsilon_{ij}$ is the random error. Dunn’s post-hoc test [41,42] for multiple comparisons of groups with Hochberg’s adjustment [43,44] was used to further examine which treatment was responsible for the differences among means of fatty acids that were statistically significant in the one-way ANOVA.

## 3. Results

### 3.1. Fatty Acid Composition of Basal and Supplementary Feeds

The fatty acid profiles of the basal (hay), control (without oil), omega-3 oil-fortified (omega-3), and whole grain (MSM) pelleted diets are shown in Table 2. All the supplementary diets were formulated to be isocaloric (metabolizable energy of 14–15 MJ/kg) and isonitrogenous (crude protein of 16.4–17.0%) with a dry matter digestibility of 83.8–87.5%. As depicted in Table 1, the omega-3 oil-infused diet had higher EPA + DHA + DPA, n-3 LC-PUFA and ALA (2.74, 19.18, and 15.39 mg/100 g, respectively), and lower total n-6 PUFA and ratios of n-6/n-3 and PUFA/SFA (28.16, 1.47, and 0.75 mg/100 g, respectively) than the control and MSM whole grain diets. The MSM whole grain diet had the highest proportions of C18:2n-6 (linoleic acid) and oleic acid (C18:1) (92.38 and 75.06 mg/100 g, respectively). The control diet had a higher n-6/n-3 ratio value of 9.08, while the basal hay diet had the highest proportion of C20:2n-6 and C20:4n-6 (arachidonic acid).

### 3.2. Fatty Acid Profile of the Longissimus thoracis et lumborum Muscle

**Figure 1:**
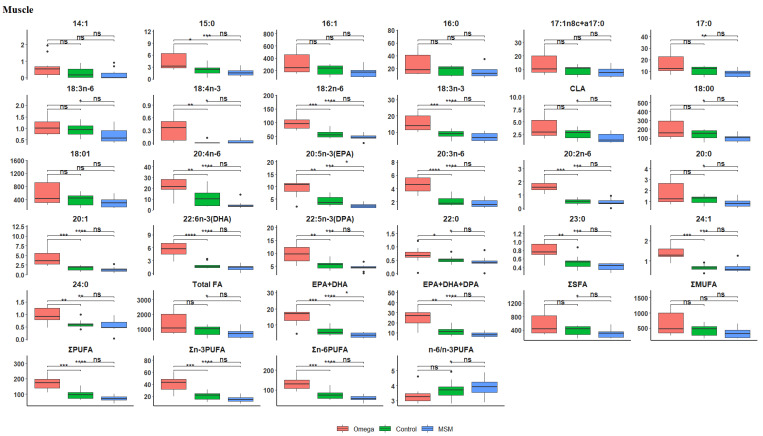
Boxplots showing the distribution of selected fatty acid composition in the muscle tissue. Each plot tested the mean fatty acid in omega-3 versus control, omega-3 versus MSM whole grain, and control versus MSM whole grain with Hochberg’s adjusted multiple comparisons. * *p* < 0.05; ** *p* < 0.01; *** *p* < 0.001; **** *p* < 0.0001; ns, not significant (*p* > 0.05).

The fatty acid composition of the *longissimus thoracis et lumborum* muscle tissue is presented in Table 3. The omega-3 oil diet produced lamb muscles with the highest contents of n-3 LC-PUFA, DHA, EPA, DPA, C18:3n-3, C18:1, C18:0 (stearic acid), total SFA, MUFA, and PUFA/SFA ratio. The MSM whole grain diet produced muscles with the highest n-6/n-3 PUFA ratio. A boxplot of Hochberg’s adjusted multiple comparisons of significant differences between the treatment groups in muscle fatty acid profiles are depicted in Figure 1, where the omega-3 diet consistently shows a significantly higher fatty acid concentration than the control and MSM whole grain diets.

### 3.3. Fatty Acid Content of Liver

**Figure 2:**
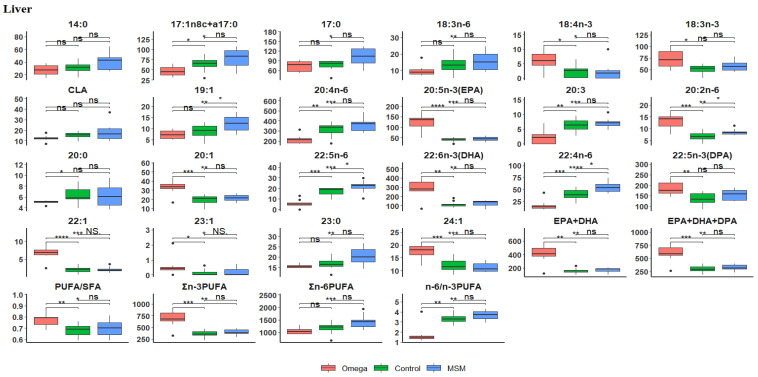
Boxplots showing the distribution of selected fatty acids composition in the liver. Each plot tested the mean fatty acid in omega-3 versus control, omega-3 versus MSM whole grain, and control versus MSM whole grain with Hochberg’s adjusted multiple comparisons. * *p* < 0.05; ** *p* < 0.01; *** *p* < 0.001; **** *p* < 0.0001; ns, not significant (*p* > 0.05).

In the liver (Table 4), it was evident that the sheer volume of total fatty acid metabolism output was greater than in the muscle, kidney, and heart. The omega-3 oil diet had significantly higher CLA (*p* < 0.0346), EPA + DHA (*p* < 0.0000), EPA + DHA + DPA (*p* < 0.0002), PUFA/SFA (*p* < 0.0365), n-3 LC-PUFA (*p* < 0.0004), n-6 PUFA (*p* < 0.0008), and n-6/n-3 PUFA ratio (*p* < 0.0000) than the other treatment groups. A boxplot of Hochberg’s adjusted multiple comparisons of significant differences between the treatment groups in liver fatty acid profiles are depicted in Figure 2, where the omega-3 oil diet maintained a significantly higher fatty acid content than the control and MSM whole grain diets. However, the compositions of C14:0, C17:1n8c + a17:0, C18:3n-6, C19:1, C20:4n-6, C20:3, CLA, C22:5n-6, C22:4n-6, C23:0, n-6 PUFA, and n-6/n-3 PUFA ratio in the MSM whole grain diet were higher than in the control and omega-3 oil diets.

### 3.4. Fatty Acid Profile of the Kidney

**Figure 3:**
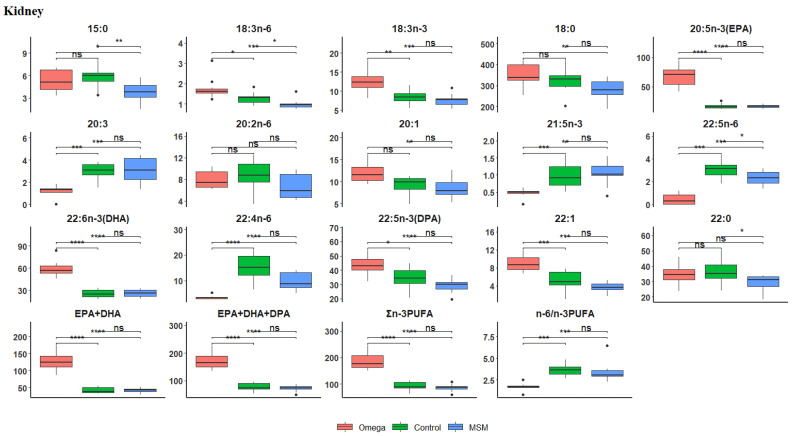
Boxplots showing the distribution of selected fatty acids composition in the kidney. Each plot tested the mean fatty acid in omega-3 versus control, omega-3 versus MSM whole grain and control versus MSM whole grain with Hochberg’s adjusted multiple comparisons. * *p* < 0.05; ** *p* < 0.01; *** *p* < 0.001; **** *p* < 0.0001; ns, not significant (*p* > 0.05).

The fatty acid profile of the kidney is presented in Table 5. Within the n-3 LC-PUFA, the contents of ALA, EPA, DHA, DPA, EPA + DHA, and EPA + DHA + DPA were greater in the omega-3 oil diet than in the control and MSM whole grain diets. The overall increase in the contents of 18:3 n-3 and its long chain metabolites remained statistically significant. A boxplot of Hochberg’s adjusted multiple comparisons of significant differences between the treatment groups in kidney fatty acid profiles are depicted in Figure 3, where the omega-3 oil diet maintained a significantly higher fatty acid content than the control and MSM whole grain diets. However, the control diet had the highest contents of C22:0, C20:2n-6, C22:5n-6, C22:4n-6, and n-6/n-3 PUFA ratio, while the MSM whole grain diet led in C20:0 and C21:5n-3 contents.

### 3.5. Fatty Acid Profile of the Heart

**Figure 4:**
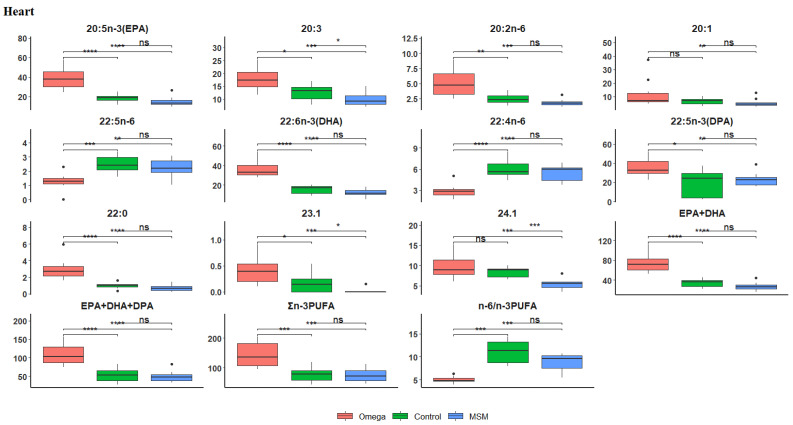
Boxplots showing the distribution of selected fatty acids composition in the heart. Each plot tested the mean fatty acid in omega-3 versus control, omega-3 versus MSM whole grain, and control versus MSM whole grain with Hochberg’s adjusted multiple comparisons. * *p* < 0.05; ** *p* < 0.01; *** *p* < 0.001; **** *p* < 0.0001; ns, not significant (*p* > 0.05).

Table 6 shows the fatty acid contents of the heart. The hearts from lambs on the omega-3 oil diet had the highest ALA, EPA, C20:2n-6, EPA + DHA, EPA + DHA + DPA, n-3 LC-PUFA, DHA, and DPA contents and lowest n-6/n-3 PUFA ratio than in the control and MSM whole grain diets. However, the hearts of lambs fed the control diet had the highest contents of C23:0, C22:0m and C22:4n-6, while those on the MSM whole grain diet had the highest C20:3 and C21:5 n-3 contents. As shown in Figure 4, a boxplot of Hochberg’s adjusted multiple comparisons of significant differences between the treatment groups in the heart fatty acid profiles shows that the omega-3 oil diet maintained a significantly higher EPA, DHA, EPA + DHA, EPA + DHA + DPA, and ∑n-3PUFA content than the control and MSM whole grain diets, while the control diet had higher C22:4n-6 content and n-6/n-3 PUFA ratio than both omega-3 oil and MSM whole grain diets.

## 4. Discussion

A prospective cohort study of men in the USA [45] and a cross-sectional survey of Korean adults [46] both reinforced the need to enhance a healthier composition of red meat among consumers to minimize the dietary risks of coronary heart disease, cardiometabolic, and cancer mortality burdens. Previous studies [22,47,48,49,50,51] had established that the fatty acid profiles of muscles and organs can be modified by dietary supplementation with n-3 LC-PUFA, leading to higher human health beneficial EPA + DHA + DPA and lower n-3/n-6 ratio [52]. It is important to strike a balance between attaining higher total PUFA deposition in the muscle and oxidative stability [53] because meat color, flavor, nutritional value, shelf-life, and overall consumer acceptance can be compromised by lipid oxidation [54,55]. Therefore, in this study, the time-tested and oxidatively stable omega-3 oil infused pellets previously reported in Le et al. [49] were utilized in the comparative analysis with non-oil and whole grain pellets.

The level of incorporation and abundance of both monounsaturated (MUFA) and polyunsaturated (PUFA) fatty acids in the muscle is consistent with previously reported intramuscular fatty acid compositions [56,57]. A review of pre-clinical and human trials with conjugated linoleic acid CLA (C18:2n-6) revealed positive effects on cancer, obesity, and atherosclerosis [58,59], and the muscle in the present study had significantly incorporated levels of CLA and ALA (C18:3n-3). ALA is the precursor for the synthesis of n-3 LC-PUFA through desaturation and/or chain-elongation by desaturase and elongase enzymes [60,61]. In the present study, it was evident that supplementation of lambs with omega-3 oil-fortified pellets increased the muscle contents of ALA by two-to three-folds, which translated into higher contents of EPA + DHA + DPA than in muscles from the control and whole grain diets. Furthermore, the high and significant levels of C20:4n-6 in the muscle could be a result of LA being subjected to elongation and desaturation by delta-5 and delta-6 desaturases and elongase enzymes [62], since all dietary treatments favored de novo fatty acid synthesis due to high levels of C18:1 originating from elongation and desaturation of C16:0 into palmitoleic and C18:0 into oleic acids [63,64,65]. In terms of dietary n-6/n-3 PUFA ratio, the human nutrition guidelines recommend an ideal value of not more than 4 [66], because n-3 PUFA plays an anti-inflammatory role [67], while n-6 PUFA exerts pro-inflammatory effects [68] in asthma and rheumatoid arthritis [69] and increased risk of cancer [70]. This study clearly demonstrated that supplementing lambs with omega-3 oil is an excellent nutritional strategy for lowering the n-6/n-3 ratio in the muscle for a healthier meat.

The liver is responsible for the metabolism, uptake, and dissemination of lipids through free fatty acids, lipoproteins, and *de novo* lipogenesis [71]. It plays a role in mitochondrial fatty acid β-oxidation and facilitates key catabolic pathways in hepatocytes [71] and ruminant meat production [72]. In the liver of lambs supplemented with omega-3 oil, the significantly higher proportions of EPA + DHA + DPA compared to the control diet equivalent to three-folds of what is obtainable in the kidney and heart (Table 5 and Table 6) in the present study, align with earlier reports [35,49,51]. These figures are well above the Food Standards of Australia and New Zealand recommendations of 30 mg/100 g as ‘source’ and 60 mg/100 g as ‘good source’ levels [49], and are in agreement with previous studies showing that the lamb liver is one of the richest sources of EPA + DHA + DPA, comprising approximately 185 mg/100 g of hepatocytes from omega-3 oil supplemented diet [73]. The liver levels of CLA were also high in lambs supplemented with omega-3 oil, and given its reported immuno-modulatory, anti-obesity, and anti-carcinogenic properties [74,75], the liver could be considered a healthy product. The major synthesis pathway of the CLA isomer, rumenic acid, in the tissues of ruminants is primarily from the biohydrogenation of ALA into vaccenic acid (C18:1n-7) catalyzed by reductase enzymes [76,77]. The variation patterns and ranges in liver fatty acid profiles in the present study were similar to previously reported findings [78,79,80,81,82,83]. The liver has also been reported to have a high nutrient content of essential amino acids, fatty acids, iron, zinc, magnesium, selenium, calcium, vitamins B1, B6, B12, and folic acid [84,85] and may explain why duck liver attracts a price premium and is consumed in France [86].

Mashek and Coleman [87] showed that the kidney plays a role in cellular fatty acid uptake and contributes to metabolism, with strong suggestions that the metabolic demand for fatty acids is a major driving force governing fatty acid uptake in the kidney. Although the mechanism is unknown, it appears that converting fatty acids to acyl-CoAs and downstream metabolic intermediates increases cellular fatty acid uptake, probably by limiting efflux [87]. Hagve et al. [88] demonstrated that increasing levels of n-3 fatty acids in membranes affect the uptake and intracellular metabolism of fatty acids as well as membrane fluidity in the kidney. Evidence from both gain and loss-of-function experiments indicate that fatty acid uptake can be modulated by activation at both the plasma membrane and internal organ sites by intracellular fatty acid binding proteins, and by enzymes in synthetic or degradative metabolic pathways [87]. In a study of long-chain polyunsaturated fatty acids metabolism in kidney cells, Liabo et al. [89] argued that only little is known about the metabolism of fatty acids in the kidney, because it is controversial whether the kidney possesses the ability to desaturate long-chain fatty acids or kidney cells are dependent on pre-formed polyunsaturated fatty acids transported from the liver. However, they concluded that the kidney, at least in part, must obtain its C-20 and C-22 fatty acids from circulation, while the active delta5-desaturase suggests that pre-formed C-20 fatty acids can be converted to more unsaturated homologues in the kidney. This could probably explain the significant increases in the contents of C18:3n-3 and its long chain ALA, EPA, DHA, and DPA metabolites being greater in the kidney of lambs supplemented with omega-3 oil than in the control and MSM whole grain diets observed in the present study (Table 4 and Figure 3).

Schaap et al. [90] reported that long-chain fatty acids are important fuel molecules in the heart, because their oxidation in the mitochondria provides the bulk of the energy required for cardiac functioning. However, the cellular transport of fatty acids in aqueous solutions is impaired due to low solubility. To circumvent this hurdle, cardiac tissues contain several fatty acid-binding proteins (FABP) capable of non-covalently binding to fatty acids, thus facilitating both cellular uptake and intracellular transport of fatty acids. The majority of fatty acids taken up by the heart seems to pass the sarcolemma through a carrier-mediated translocation mechanism consisting of one or more membrane-associated FABP [81]. Perhaps the observed significant differences between the treatment groups in the heart fatty acid profiles in the present study where the omega-3 oil diet maintained a significantly higher EPA, DHA, EPA + DHA, EPA + DHA + DPA, and ∑n-3PUFA content than the control and MSM whole grain diets, could probably be indicative of higher activities of the FABP in intracellular transport and cellular uptake of long chain fatty acids.

## 5. Conclusions

This is the first study that evaluated and compared the fatty acid profiles in the tissues and organs of TAW MARGRA lambs raised in a feedlot production system in response to dietary supplementation with or without fortification with omega-3 oil. It was primarily to shed some light on n-3 LC-PUFA metabolism in the *Longissimus thoracis et lumborum* muscle, heart, kidney, and liver of lot-fed TAW MARGRA lambs in response to dietary supplementation with omega-3 oil. The findings suggest that dietary manipulation can be utilized to improve the fatty acid content and nutritional value of muscle and organs of TAW MARGRA lambs to meat that more closely meets nutritional guidelines of higher levels of health-beneficial n-3 LC-PUFA. The data clearly portray the liver, kidney, and heart of TAW MARGRA lambs with highest contents of the healthiest omega-3 fatty acids well beyond the FSANZ ‘good source’ levels. Therefore, the hypothesis that *fortifying feedlot pellets with omega-3 oil will enhance the human health beneficial n-3 LC-PUFA composition of edible lamb muscle tissue and organs* holds, and is worthy of acceptance.

## Figures and Tables

**Table 1:** Nutrient composition of the control, n-3 LC PUFA, MSM whole grain, and hay feeds ^#^.

Nutrient Composition (%DM)	Experimental and Basal Diets			
	Control	Omega-3	MSM Whole Grain	Hay
Dry Matter (DM)	90.8	91.7	90.3	93.4
Moisture	9.2	8.3	9.7	6.6
Acid Detergent Fiber (ADF)	7.6	8.2	6.3	39.4
Neutral Detergent Fiber (NDF)	23.8	23.0	21.8	60.9
Crude Protein (CP)	16.9	17.0	16.4	7.5
Ash	7.8	8.2	7.2	8.1
Ether Extract (EE)	6.1	10.3	6.0	3.3
Metabolizable Energy (ME) MJ/kg	14.1	15.1	14.4	8.3
Dry Matter Digestibility (DMD)	84.9	83.8	87.5	46.8
Digestible Organic Matter (DOMD)	83.7	82.6	86.2	47.1

^#^ DOMD, Digestible organic matter in the dry matter; TDN, %Total digestible nutrients (as % of DM) = 82.38 − (0.7515 × ADF [% of DM]); ME, Metabolizable energy (DE [Mcal/kg] = %TDN × 0.01 × 4.4) where DE is digestible energy, which was converted as ME = (DE (Mcal/kg) × 0.82) × 4.185.

**Table 2:** Fatty acid composition (mg/100 g) of basal and supplementary diets ^#^.

Fatty Acid	Omega-3	Control	MSM Whole Grain	Basal Hay
13:0	0.00	0.00	0.01	0.00
14:1	0.00	0.00	0.00	0.00
14:0	2.23	0.45	0.25	0.33
15:0	2.62	0.36	0.30	0.21
16:1	1.02	0.79	1.05	1.88
16:0	35.96	27.19	30.52	12.53
17:1n8c + a17:0	0.64	0.27	0.25	0.18
17:0	1.09	0.33	0.30	0.13
18:3n6	0.32	0.00	0.00	0.24
18:4n3	0.32	0.00	0.00	0.20
18:2n6 (LA)	25.25	81.75	92.38	30.02
18:3n3 (ALA)	15.39	8.84	11.21	6.13
CLA	0.65	0.10	0.29	0.00
18:0	8.25	4.59	4.13	2.74
18:1	31.92	57.34	75.06	51.23
19:1	0.00	0.04	0.06	0.04
20:4n6 (ARA)	0.00	0.00	0.00	0.53
20:5n3 (EPA)	0.22	0.18	0.23	0.00
20:3	0.00	0.00	0.01	0.00
20:3n6	0.80	0.15	0.16	0.18
20:4n3	0.30	0.00	0.00	0.02
20:2n6	0.32	0.13	0.21	0.33
20:1	1.51	1.71	2.02	1.79
20:0	2.80	0.60	0.81	0.42
21:5n3	0.43	0.00	0.00	0.00
21:0	1.17	0.16	0.08	0.00
22:5n6	0.87	0.01	0.08	0.00
22:6n3 (DHA)	1.53	0.03	0.05	0.93
22:4n6	0.60	0.08	0.05	0.00
22:5n3 (DPA)	0.99	0.00	0.17	0.22
22:1	2.39	0.47	0.34	0.25
22:0	4.63	0.44	0.60	0.23
23:1	0.64	0.06	0.00	0.00
23:0	1.72	0.01	0.14	0.02
24:1	0.94	0.25	0.28	0.14
24:0	3.54	0.40	0.54	0.15
Total FA	147.11	185.91	220.96	110.69
EPA + DHA	1.74	0.21	0.28	0.93
EPA + DHA + DPA	2.74	0.21	0.45	1.16
SFA	64.02	34.52	37.66	16.77
MUFA	35.09	60.13	78.46	55.12
PUFA	48.00	91.26	104.84	38.81
PUFA/SFA	0.75	2.64	2.78	2.31
∑n3PUFA	19.18	9.05	11.66	7.51
∑n6PUFA	28.16	82.12	92.88	31.30
n6/n3PUFA	1.47	9.08	7.97	4.17

^#^ LA, linoleic acid; ALA, α-linolenic acid; CLA, conjugated linolenic acid; ARA, arachidonic acid; EPA, eicosapentaenoic acid; DHA, docosahexaenoic acid; DPA, docosapentaenoic acid; ΣSFA, total saturated fatty acids; FA, fatty acid; ΣMUFA, total monounsaturated fatty acids; and total polyunsaturated fatty acids (ΣPUFA); ∑SFA is the sum of 14:0, 15:0, 16:0, 17:0, 18:0, 20:0, 21:0, 22:0, 23:0, 24:0; ∑MUFA is the sum of 14:1, 16:1, 17:1n-8 + a17:0, 18:1, 19:1, 20:1, 22:1, 23:1, 24:1; ∑PUFA is the sum of 18:4n-3, 18:3n-6, 18:2n-6, 18:3n-3, 20:3, 20:4n-3, 20:4n-6, 20:5n-3, 20:3n-6, 20:2n-6, 22:6n-3, 22:5n-3, 22:5n-6, 22:4n-6; ∑n-3 LC-PUFA is the sum of 18:3n-3, 18:4n-3, 20:4n-3, 20:5n-3, 22:6n-3, 22:5n-3; ∑n-6 PUFA is the sum of 18:2n-6, 18:3n-6, 20:4n-6, 20:3n-6, 20:2n-6, 22:5n-6, 22:4n-6.

**Table 3:** Fatty acid profile (mg/100 g) of *Longissimus thoracis et lumborum* muscle tissue in TAW lambs ^#^.

Fatty Acid	Omega-3	Control	MSM Whole Grain	*p*-Value
**13:0**	0.05 ± 0.11	0.03 ± 0.04	0.00 ± 0.00	0.1108
**14:1**	0.63 ± 0.65	0.31 ± 0.33	0.19 ± 0.34	0.0417
**14:0**	24.25 ± 18.62	16.80 ± 7.84	14.22 ± 8.06	0.0809
**15:1**	4.44 ± 2.36	2.26 ± 1.22	1.58 ± 0.85	0.0005
**16:0**	320.50 ± 182.90	209.83 ± 83.05	169.06 ± 79.43	0.0106
**16:1**	25.86 ± 17.40	16.93 ± 7.29	14.43 ± 8.57	0.0398
**17:1n8c + a17:0**	13.83 ± 7.99	9.40 ± 3.68	7.85 ± 3.55	0.0201
**17:0**	16.85 ± 9.12	10.80 ± 4.08	8.31 ± 3.10	0.0035
**18:3n6**	1.09 ± 0.38	0.93 ± 0.28	0.71 ± 0.31	0.0116
**18:4n-3**	0.34 ± 0.28	0.01 ± 0.04	0.03 ± 0.05	0.0006
**18:2n-6 (LA)**	99.35 ± 25.12	57.06 ± 14.44	46.89 ± 10.66	0.0000
**18:3n-3 (ALA)**	15.70 ± 5.01	8.76 ± 2.46	6.87 ± 2.64	0.0000
**CLA**	3.83 ± 2.05	2.65 ± 1.03	1.79 ± 0.93	0.0030
**18:0**	208.83 ± 119.80	139.11 ± 54.40	99.63 ± 40.82	0.0042
**18:1**	582.31 ± 364.95	395.67 ± 173.74	305.06 ± 147.93	0.0174
**19:1**	1.31 ± 0.68	0.89 ± 0.37	0.93 ± 0.51	0.1222
**20:4n-6 (ARA)**	21.87 ± 8.20	11.26 ± 8.18	4.78 ± 3.64	0.0000
**20:5n-3 (EPA)**	9.68 ± 3.68	4.25 ± 2.15	2.36 ± 1.01	0.0000
**22:3**	0.19 ± 0.31	0.21 ± 0.22	0.18 ± 0.23	0.9168
**20:3n-6**	4.61 ± 1.17	2.10 ± 0.68	1.74 ± 0.58	0.0000
**20:4n-3**	0.02 ± 0.05	0.10 ± 0.18	0.15 ± 0.24	0.0945
**20:2n-6**	1.66 ± 0.41	0.52 ± 0.20	0.45 ± 0.29	0.0000
**20:0**	1.71 ± 1.01	1.15 ± 0.40	0.82 ± 0.37	0.0051
**20:1**	4.45 ± 2.34	1.71 ± 0.63	1.30 ± 0.68	0.0001
**21:5n-3**	0.15 ± 0.13	0.14 ± 0.12	0.11 ± 0.12	0.4905
**21:0**	0.13 ± 0.22	0.04 ± 0.09	0.10 ± 0.14	0.5992
**22:5n-6**	0.27 ± 0.22	0.26 ± 0.25	0.24 ± 0.11	0.6978
**22:6n-3 (DHA)**	5.59 ± 1.63	1.85 ± 0.81	1.28 ± 0.67	0.0000
**22:4n-6**	1.10 ± 0.39	0.93 ± 0.33	0.91 ± 0.25	0.2010
**22:5n-3 (DPA)**	9.69 ± 3.36	5.48 ± 1.75	4.42 ± 1.25	0.0000
**22:0**	0.48 ± 0.27	0.34 ± 0.17	0.36 ± 0.43	0.3908
**22:1**	0.68 ± 0.31	0.52 ± 0.14	0.43 ± 0.21	0.0218
**23:1**	0.00 ± 0.00	0.00 ± 0.00	0.02 ± 0.05	0.2268
**23:0**	0.78 ± 0.18	0.52 ± 0.16	0.41 ± 0.09	0.0000
**24:1**	1.37 ± 0.33	0.66 ± 0.16	0.66 ± 0.24	0.0000
**24:0**	0.98 ± 0.29	0.61 ± 0.17	0.53 ± 0.25	0.0004
**Total FA**	1384.60 ± 766.56	904.11 ± 342.20	698.78 ± 307.88	0.0056
**EPA + DHA**	15.28 ± 5.12	6.11 ± 2.88	3.64 ± 1.49	0.0000
**EPA + DHA + DPA**	24.97 ± 8.27	11.58 ± 4.54	8.05 ± 2.56	0.0000
**∑** **SFA**	579.40 ± 333.52	381.87 ± 149.39	295.27 ± 131.95	0.0079
**∑** **MUFA**	630.23 ± 393.61	425.92 ± 185.28	330.79 ± 161.51	0.0172
**∑** **PUFA**	175.14 ± 47.59	96.53 ± 28.59	72.90 ± 19.03	0.0000
**PUFA/SFA**	0.36 ± 0.14	0.29 ± 0.12	0.27 ± 0.10	0.1210
**∑** **n-3PUFA**	41.36 ± 12.83	20.81 ± 6.43	15.38 ± 5.24	0.0000
**∑** **n-6PUFA**	133.78 ± 35.37	75.71 ± 22.91	57.51 ± 14.31	0.0000
**n-6/n-3PUFA**	3.33 ± 0.52	3.72 ± 0.63	3.87 ± 0.65	0.0499

^#^ Abbreviations as in Table 2.

**Table 4:** Fatty acid profile (mg/100 g) of the liver in TAW lambs ^#^.

Fatty Acid	Omega-3	Control	MSM Whole Grain	*p*-Value
13:0	0.24 ± 0.26	0.16 ± 0.26	0.36 ± 0.50	0.3890
14:1	0.84 ± 0.92	1.45 ± 1.21	0.55 ± 0.86	0.6411
14:0	26.95 ± 8.36	31.22 ± 8.74	40.09 ± 13.89	0.0190
15:1	19.20 ± 6.55	15.49 ± 5.47	18.81 ± 7.39	0.7370
16:0	755.15 ± 151.51	765.95 ± 134.32	906.84 ± 205.56	0.0938
16:1	66.47 ± 18.67	83.97 ± 26.54	89.50 ± 28.94	0.0783
17:1n8c + a17:0	46.43 ± 11.84	62.51 ± 16.78	78.31 ± 25.78	0.0017
17:0	73.54 ± 16.08	76.10 ± 16.51	101.28 ± 26.67	0.0143
18:3n6	9.67 ± 3.17	13.31 ± 4.89	15.45 ± 5.60	0.0160
18:4n-3	6.10 ± 3.34	2.34 ± 2.22	2.15 ± 3.18	0.0098
18:2n-6 (LA)	508.28 ± 68.59	438.04 ± 84.82	570.01 ± 99.23	0.2330
18:3n-3 (ALA)	72.78 ± 18.68	50.46 ± 9.52	58.65 ± 12.48	0.0655
CLA	12.45 ± 2.57	15.13 ± 3.13	18.05 ± 8.47	0.0346
18:0	1050.30 ± 82.46	879.11 ± 139.95	1058.24 ± 190.38	0.9770
18:1	1414.42 ± 210.44	1414.52 ± 268.81	1521.50 ± 345.97	0.4875
19:1	7.25 ± 1.88	8.93 ± 2.98	12.35 ± 3.47	0.0010
20:4n-6 (ARA)	213.71 ± 41.72	310.53 ± 69.00	368.35 ± 71.25	0.0000
20:5n-3 (EPA)	122.60 ± 36.37	40.03 ± 9.65	43.99 ± 10.29	0.0000
22:3	2.08 ± 2.23	6.35 ± 2.14	7.16 ± 1.72	0.0001
20:3n-6	58.65 ± 9.41	33.88 ± 5.74	49.26 ± 16.35	0.1700
20:4n-3	6.07 ± 0.84	5.00 ± 2.59	6.03 ± 1.75	0.9550
20:2n-6	12.96 ± 3.02	6.72 ± 1.79	8.59 ± 1.24	0.0033
20:0	33.42 ± 7.26	19.28 ± 5.06	21.45 ± 3.60	0.0652
20:1	5.02 ± 0.28	6.25 ± 1.61	6.38 ± 1.97	0.0007
21:5n-3	1.58 ± 0.71	3.27 ± 2.79	2.76 ± 2.20	0.2770
21:0	0.37 ± 0.21	0.28 ± 0.29	0.51 ± 0.26	0.2643
22:5n-6	5.23 ± 3.89	17.08 ± 3.63	21.53 ± 5.93	0.0000
22:6n-3 (DHA)	286.77 ± 95.79	116.09 ± 30.92	124.56 ± 30.70	0.0001
22:4n-6	16.44 ± 9.99	39.84 ± 11.41	54.47 ± 11.01	0.0000
22:5n-3 (DPA)	185.12 ± 30.84	133.09 ± 29.99	154.08 ± 24.89	0.0515
22:0	6.78 ± 1.87	2.05 ± 0.87	2.12 ± 0.74	0.0000
22:1	9.76 ± 0.84	8.20 ± 1.11	9.31 ± 1.81	0.5057
23:1	0.56 ± 0.57	0.14 ± 0.22	0.17 ± 0.27	0.0504
23:0	15.48 ± 0.82	16.69 ± 2.77	20.36 ± 4.07	0.0011
24:1	17.44 ± 2.66	11.81 ± 2.62	11.14 ± 1.71	0.0000
24:0	16.10 ± 1.00	15.49 ± 2.52	17.48 ± 2.82	0.2397
Total FA	5085.02 ± 632.20	4650.60 ± 806.74	5421.65 ± 1044.59	0.5016
EPA + DHA	409.37 ± 127.95	156.12 ± 37.43	168.54 ± 35.69	0.0000
EPA + DHA + DPA	594.49 ± 153.14	289.22 ± 63.49	322.62 ± 56.93	0.0002
∑SFA	1972.10 ± 229.59	1814.93 ± 295.51	2179.67 ± 431.38	0.2769
∑MUFA	1593.04 ± 242.26	1604.51 ± 317.62	1736.91 ± 404.01	0.4181
∑PUFA	1519.88 ± 191.39	1231.16 ± 228.83	1505.07 ± 261.64	0.8519
PUFA/SFA	0.77 ± 0.06	0.68 ± 0.06	0.70 ± 0.07	0.0365
∑n-3PUFA	682.48 ± 170.17	356.63 ± 72.64	399.37 ± 68.16	0.0004
∑n-6PUFA	1045.88 ± 117.45	1167.97 ± 233.56	1452.52 ± 275.64	0.0008
n-6/n-3PUFA	1.69 ± 0.83	3.30 ± 0.43	3.66 ± 0.49	0.0000

^#^ Abbreviations as in Table 2.

**Table 5:** Fatty acid profile (mg/100 g) of the kidney in TAW lambs ^#^.

Fatty Acid	Omega-3	Control	MSM Whole Grain	*p*-Value
**13:0**	0.22 ± 0.21	0.16 ± 0.22	0.28 ± 0.22	0.5721
**14:1**	0.1 ± 0.14	0.05 ± 0.15	0.04 ± 0.07	0.2296
**14:0**	7 ± 2.29	6.96 ± 2.3	5.42 ± 1.27	0.0886
**15:1**	5.33 ± 1.39	5.73 ± 1.45	3.72 ± 1.33	0.0217
**16:0**	317.14 ± 52.86	339.32 ± 63.53	283.75 ± 48.67	0.2055
**16:1**	10.57 ± 2.87	11.21 ± 2.56	11.39 ± 2.94	0.5105
**17:1n8c + a17:0**	12.44 ± 1.91	14.29 ± 3.33	12.04 ± 2.77	0.7594
**17:0**	25.4 ± 3.66	28.7 ± 7.39	23.32 ± 5.09	0.4353
**18:3n6**	1.75 ± 0.54	1.28 ± 0.28	0.98 ± 0.25	0.0001
**18:4n-3**	0.00 ± 0.00	0.04 ± 0.12	0.01 ± 0.03	0.7995
**18:2n-6 (LA)**	281.42 ± 66.94	280.63 ± 73.32	249.57 ± 56.92	0.2849
**18:3n-3 (ALA)**	12.43 ± 3.02	8.4 ± 1.62	7.63 ± 1.61	0.0001
**CLA**	3.5 ± 0.82	3.78 ± 0.85	3.11 ± 1.14	0.3653
**18:0**	351.69 ± 51.59	327.73 ± 62.89	279.14 ± 47.83	0.0054
**18:1**	320.27 ± 53.92	314.51 ± 56.7	293.23 ± 58.49	0.2854
**19:1**	2.17 ± 0.44	3.84 ± 1.28	2.9 ± 1.01	0.1645
**20:4n-6 (ARA)**	169.81 ± 27.05	246.86 ± 57.97	209.05 ± 37.93	0.0940
**20:5n-3 (EPA)**	69.1 ± 17.67	16.87 ± 4.66	16.57 ± 2.71	0.0000
**20:3**	1.1 ± 0.62	2.98 ± 0.75	3.06 ± 1.12	0.0001
**20:3n-6**	19.6 ± 3.26	17.41 ± 5.3	12.88 ± 3.59	0.0010
**20:4n-3**	1.95 ± 0.45	1.91 ± 0.92	2.54 ± 1.71	0.2627
**20:2n-6**	7.9 ± 1.65	8.77 ± 2.68	6.62 ± 2.3	0.2320
**20:0**	5.35 ± 1.12	5.84 ± 1.13	4.91 ± 0.85	0.3655
**20:1**	11.88 ± 2.14	9.14 ± 2.2	8.52 ± 2.19	0.0020
**21:5n-3**	0.48 ± 0.13	0.99 ± 0.38	1.04 ± 0.35	0.0006
**21:0**	0.69 ± 0.13	0.82 ± 0.16	0.65 ± 0.13	0.5464
**22:5n-6**	0.44 ± 0.49	3.03 ± 0.73	2.33 ± 0.62	0.0003
**22:6n-3 (DHA)**	58.76 ± 10.76	25.17 ± 5.59	25.3 ± 5.21	0.0000
**22:4n-6**	3.52 ± 0.76	15.04 ± 5.17	9.88 ± 3.32	0.0133
**22:5n-3 (DPA)**	43.39 ± 6.14	33.76 ± 8.04	29.14 ± 4.96	0.0000
**22:0**	35.02 ± 6.6	36.52 ± 8.02	29.06 ± 5.3	0.0000
**22:1**	9.01 ± 1.71	5.12 ± 2.16	3.62 ± 1.08	0.0659
**23:1**	0.56 ± 0.23	0.96 ± 0.34	0.8 ± 0.28	0.1012
**23:0**	8.84 ± 1.35	9.96 ± 1.97	7.94 ± 1.71	0.2826
**24:1**	30.55 ± 5.54	33.12 ± 7.47	32.73 ± 7.26	0.4763
**24:0**	34.42 ± 5.47	37.53 ± 7.62	31.21 ± 6.79	0.3120
**Total FA**	1499.2 ± 208.97	1515.42 ± 308.98	1322.14 ± 193.73	0.1155
**EPA + DHA**	127.86 ± 26.2	42.04 ± 9.14	41.87 ± 7.28	0.0000
**EPA + DHA + DPA**	171.26 ± 29.76	75.8 ± 15.82	71.01 ± 10.98	0.0000
**∑** **SFA**	439.41 ± 67.09	471.55 ± 89.38	390.25 ± 66.92	0.1743
**∑** **MUFA**	385.11 ± 61.94	377.95 ± 69.57	353.22 ± 71.1	0.2933
**∑** **PUFA**	674.69 ± 91.83	665.92 ± 156.17	578.66 ± 79.86	0.0698
**PUFA/SFA**	1.54 ± 0.13	1.4 ± 0.12	1.51 ± 0.29	0.7367
**∑** **n-3PUFA**	187.22 ± 32.15	90.12 ± 18.16	85.28 ± 13.7	0.0000
**∑** **n-6PUFA**	318.13 ± 70.45	329.97 ± 86.61	285.38 ± 60.57	0.3264
**n-6/n-3PUFA**	1.74 ± 0.42	3.65 ± 0.64	3.44 ± 1.14	0.0003

^#^ Abbreviations as in Table 2.

**Table 6:** Fatty acid profile (mg/100 g) of the heart in TAW lambs ^#^.

Fatty Acid	Omega-3	Control	MSM Whole Grain	*p*-Value
13:0	0.14 ± 0.35	0.02 ± 0.07	0.03 ± 0.1	0.2533
14:1	0.51 ± 0.35	0.61 ± 0.3	0.36 ± 0.47	0.3935
14:0	45.97 ± 95.07	13.63 ± 9.65	23.69 ± 32.81	0.3994
15:1	11.42 ± 20.53	4.75 ± 2.84	6.68 ± 5.79	0.3391
16:0	539.22 ± 573.68	402.04 ± 132.98	389.7 ± 233.75	0.3616
16:1	59.06 ± 91.19	34.61 ± 18.23	41.34 ± 35.23	0.4919
17:1n8c + a17:0	33.28 ± 40.65	21.73 ± 10.42	26.43 ± 18.68	0.5655
17:0	57.87 ± 82.96	37.03 ± 17.28	41.53 ± 29.75	0.4822
18:3n6	2.81 ± 1.01	2.55 ± 0.55	2.24 ± 0.57	0.0919
18:4n-3	0.31 ± 0.46	0.15 ± 0.29	0.06 ± 0.12	0.0823
18:2n-6 (LA)	543.33 ± 141.44	591.85 ± 100.17	466.72 ± 122.48	0.1901
18:3n-3 (ALA)	31.32 ± 26.24	18.55 ± 7.28	22.03 ± 15.47	0.2636
CLA	709.39 ± 866.87	492.75 ± 233.75	459.86 ± 332.73	0.3144
18:0	862.44 ± 1163.49	557.84 ± 334.03	601.47 ± 462.38	0.4367
18:1	9.67 ± 10.06	6.03 ± 2.24	5.68 ± 3	0.1578
19:1	3.32 ± 3.49	2.59 ± 1.28	3.41 ± 2.54	0.9383
20:4n-6 (ARA)	128.74 ± 35.44	166.3 ± 33.44	143.74 ± 48.1	0.4257
20:5n-3 (EPA)	38.59 ± 11.44	18.01 ± 3.9	14.87 ± 4.96	0.0000
22:3	1.6 ± 0.83	3.05 ± 0.79	2.23 ± 0.62	0.1398
20:3n-6	18.01 ± 4.67	12.78 ± 3.01	9.71 ± 2.57	0.0000
20:4n-3	0.22 ± 0.28	0.48 ± 0.72	0.11 ± 0.24	0.6342
20:2n-6	5.12 ± 2.19	2.45 ± 0.87	1.82 ± 0.6	0.0000
20:0	6.11 ± 8.08	4.76 ± 2.14	4.27 ± 2.66	0.4158
20:1	11.82 ± 10.49	6.45 ± 2.36	5.24 ± 3.19	0.0301
21:5n-3	0.29 ± 0.55	0.62 ± 0.32	0.39 ± 0.26	0.6112
21:0	0.62 ± 0.74	0.48 ± 0.13	0.61 ± 0.46	0.9544
22:5n-6	1.26 ± 0.58	2.49 ± 0.57	2.21 ± 0.64	0.0045
22:6n-3 (DHA)	36.74 ± 9.42	15.37 ± 4.1	11.99 ± 3.73	0.0000
22:4n-6	2.91 ± 0.89	6.06 ± 1.29	5.5 ± 1.16	0.0003
22:5n-3 (DPA)	34.82 ± 9.38	19.03 ± 14.44	23 ± 6.92	0.0301
22:0	2.95 ± 1.25	0.97 ± 0.33	0.69 ± 0.38	0.0000
22:1	4.47 ± 2.07	5.03 ± 1.97	3.72 ± 1.83	0.4011
23:1	0.43 ± 0.28	0.17 ± 0.19	0.02 ± 0.05	0.0000
23:0	4.58 ± 1.46	6.38 ± 1.24	4.43 ± 1.42	0.8409
24:1	10.03 ± 3.11	8.43 ± 1.31	5.59 ± 1.55	0.0000
24:0	4.19 ± 1.24	4.65 ± 1.82	3.72 ± 1.03	0.4712
Total FA	3223.54 ± 3074.77	2470.68 ± 880	2335.08 ± 1176.42	0.3140
EPA + DHA	75.32 ± 20.32	33.38 ± 7.73	26.86 ± 8.05	0.0000
EPA + DHA + DPA	110.14 ± 28.19	52.41 ± 18.48	49.86 ± 14.91	0.0000
∑SFA	1384 ± 1645.79	971.52 ± 396.97	938.25 ± 632.49	0.3409
∑MUFA	983.83 ± 1309.31	633.39 ± 366.3	684.53 ± 519.99	0.4274
∑PUFA	855.71 ± 225.88	865.77 ± 149	712.3 ± 183.06	0.1016
PUFA/SFA	1 ± 0.42	0.98 ± 0.25	0.99 ± 0.48	0.9636
∑n-3PUFA	143.88 ± 41.03	75.26 ± 23.68	74.68 ± 23.06	0.0000
∑n-6PUFA	711.84 ± 189.55	790.5 ± 130.94	637.62 ± 168.09	0.3408
n-6/n-3PUFA	5.01 ± 0.68	11.12 ± 2.43	8.82 ± 1.92	0.0041

^#^ Abbreviations as in Table 2.

## Data Availability

Data available upon reasonable request from authors.
